# Delineating and Analyzing Locality-Level Determinants of Cholera, Haiti

**DOI:** 10.3201/eid2701.191787

**Published:** 2021-01

**Authors:** Karolina Griffiths, Kenny Moise, Martine Piarroux, Jean Gaudart, Samuel Beaulieu, Greg Bulit, Jean-Petit Marseille, Paul Menahel Jasmin, Paul Christian Namphy, Jean-Hugues Henrys, Renaud Piarroux, Stanislas Rebaudet

**Affiliations:** Aix Marseille University, Marseille, France (K. Griffiths, J. Gaudart, S. Rebaudet); Université Quisqueya, Port-au-Prince, Haiti (K. Moise, J.-H. Henrys);; Centre d’épidémiologie et de santé Publique des armées, Marseille (M. Piarroux);; UNICEF, Kinshasa, Democratic Republic of the Congo (S. Beaulieu);; UNICEF, New York, New York, USA (G. Built); Direction Nationale de l’Eau Potable et de l’Assainissement, Hinche, Haiti (J.-P. Marseille);; Ministère de la Santé Publique et de la Population, Hinche (P.M. Jasmin);; Direction Nationale de l’Eau Potable et de l’Assainissement, Petion-Ville, Haiti (P.C. Namphy);; Sorbonne Université, Paris, France (R. Piarroux)

**Keywords:** Vibrio cholerae O1, cholera, spatial analysis, Haiti, epidemiology, communicable disease prevention and control, risk factors, bacteria, enteric infections, waterborne infections, foodborne infections

## Abstract

Centre Department, Haiti, was the origin of a major cholera epidemic during 2010–2019. Although no fine-scale spatial delineation is officially available, we aimed to analyze determinants of cholera at the local level and identify priority localities in need of interventions. After estimating the likely boundaries of 1,730 localities by using Voronoi polygons, we mapped 5,322 suspected cholera cases reported during January 2015–September 2016 by locality alongside environmental and socioeconomic variables. A hierarchical clustering on principal components highlighted 2 classes with high cholera risk: localities close to rivers and unimproved water sources (standardized incidence ratio 1.71, 95% CI 1.02–2.87; p = 0.04) and urban localities with markets (standardized incidence ratio 1.69, 95% CI 1.25–2.29; p = 0.0006). Our analyses helped identify and characterize areas where efforts should be focused to reduce vulnerability to cholera and other waterborne diseases; these methods could be used in other contexts.

Haiti experienced a large and lasting cholera epidemic that began in October 2010. The epidemic originated in Centre Department in a hamlet hosting a camp of United Nations peacekeepers, then contaminated the Artibonite River and coastal plain (*1*,*2*). During 2010–2019, >820,000 suspected cholera cases and 9,792 deaths were reported (*3*). However, since February 2019 no suspected deaths have occurred, and no positive test results have been reported from >1,000 samples collected from patients with acute watery diarrhea.

Fecal contamination of water, food, or hands by toxigenic strains of the bacterium *Vibrio cholerae* O1 is the common mode of cholera transmission. The National Plan for the Elimination of Cholera, launched by the Ministry of Public Health and Population (Ministère de la Santé Publique et de la Population [MSPP]) in Haiti and co-authored by Haiti’s National Drinking Water and Sanitation Directorate (La Direction Nationale de l’Eau Potable et de l’Assainissement [DINEPA]), is now in its third phase, focusing on reinforcing access to clean drinking water and sanitation (*4*,*5*). Reducing population vulnerability to cholera and other waterborne diseases at a community level is vital. However, with the constraints of limited resources, the most pertinent at-risk geographic zones must be prioritized for sustainable water sanitation and hygiene (WaSH) interventions.

Previous studies have performed spatial analyses at communal levels, but data at a finer scale are limited. High-resolution mapping is needed to understand the heterogeneous transmission patterns and to adapt specific intervention strategies at the community level (*6*–*9*). Although a previous study by Allan et al. (*6*) provided maps of the origin of cholera patients at a subcommunal level for the neighboring Artibonite Department, these maps are still at a relatively large scale, demonstrating communal sections hosting 10,000–30,000 persons. That study reported neighboring sections within the same commune with clear differences in relative risk for cholera in a mosaic pattern, highlighting the need for further locality-level investigation to guide WaSH interventions. However, the mapping of rural localities in Haiti is sparse and no spatial delineation has been established, presenting a challenge in identifying microspots with recurrent cases and analyzing associated factors (*6*). A previous study collected fine-scale spatial data in Haiti by using spatial videos for cholera investigations; however, it was limited to intraurban areas and did not include case data (*10*).

Previous studies highlight the challenges in collecting fine-scale spatial data in Haiti, with limited official information on informal settlements (*10*–*12*). Our objective was to analyze the spatial determinants of cholera and to identify the priority localities in need of prevention interventions in the Centre Department in Haiti. We chose Centre Department because it was at the origin of the epidemic and had a high incidence of cholera that persisted for several years.

## Methods

### Study Design and Setting

**Figure 1:**
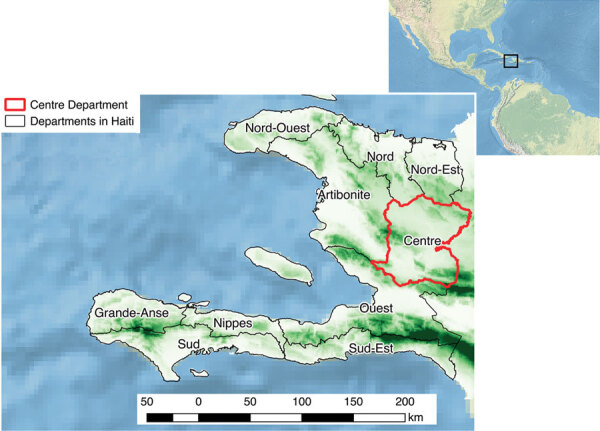
Topographical map of Haiti and its departments, highlighting Centre Department (red outline). Altitude increases from light green to dark green. Inset shows Haiti in relation to neighboring continents.

**Figure 2:**
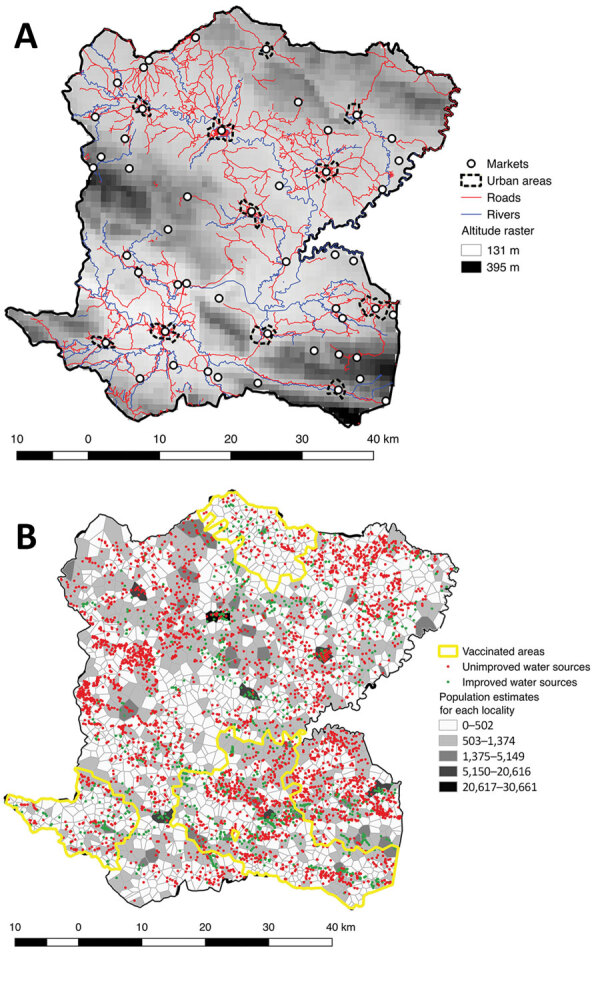
Geographic and demographic details of Centre Department, Haiti. A) Outline of the department’s 12 communes, principal urban areas, altitude raster, rivers, roads, and position of markets. B) Polygon outline and estimated population for each locality, positions of unimproved and improved water sources, and vaccinated areas.

We conducted an observational, ecologic study at the locality (hamlet) level in the Centre Department, Haiti (Figure 1), which covers an area of 3,487 km^2^, is >80% rural, and in 2015 had a population of ≈746,236 (*13*). Centre is administratively subdivided into 12 civil townships, known as communes, each of which has an urban area (Figure 2, panel A). Each commune is further subdivided into communal sections, the smallest official administrative unit, which include several hundred localities. Localities are groups of residences and are the smallest spatial unit culturally used to define the place of residence for rural populations. However, the number, spelling, and delimitations of rural localities are not officially established, and only estimated geolocalization and populations are available (*14*). 

The largest towns in Centre are Hinche and Mirebalais. Centre has a mean altitude of 447 m (range 84–1,820 m) and is situated northeast of the Montagnes Noires and Montagnes de Trou d’Eau mountain ranges and south of the Massif du Nord. Hundreds of rivers and streams provide natural water sources in the department.

### Materials

We obtained the deidentified case list of suspected cholera cases in Centre during January 2015–September 2016 from the MSPP. The case list provided the locality of residence for each patient and was collected by the Health Departmental Directorate of the MSPP to guide case-area targeted interventions conducted by rapid response teams (*15*). All patient identifiers were removed previously.

The mapping of rural localities in Haiti is incomplete (*6*). We collected point global positioning system (GPS) coordinates for each locality from field visits by mobile response teams, authors’ field visits, or satellite photos and from geographic repositories, including Haitian Institute of Statistics and Computer Science (http://ihsi.ht/publication_cd_atlas.htm), Index Mundi (https://www.indexmundi.com), OpenStreetMap (https://www.openstreetmap.org), Google Earth (https://earth.google.com), and Google Maps (https://www.google.fr/maps). We verified GPS coordinates against official data from Centre National d’Information Geospatiale (CNIGS), when available. Further data validation was performed by using computerized participatory mapping techniques to integrate spatial information from local community stakeholders in each commune, either individually or in small groups. These stakeholders included local healthcare professionals from mobile response teams, WaSH technicians, and local government personnel. We checked and unified locality names and used satellite imagery–assisted visual mapping techniques to validate the estimated GPS coordinates. This process ensured coherence with local knowledge of the terrain and identify inconsistencies, which was vital for rural areas without road access.

As part of a collaborative project financed by World Vision (https://www.worldvision.org), DINEPA and the nongovernmental partner organization Haiti Outreach (https://www.haitioutreach.org) collected an inventory of water sources and provided GPS coordinates by visiting each water point and validating data with local partners (*16*) (Figure 2, panel B). Water sources were classified as improved or unimproved according to the Joint Monitoring Program for Water Supply Sanitation and Hygiene (*17*,*18*).

We obtained the location of rivers, roads, and altitude for Centre from CNIGS (Figure 2, panel A). We obtained history of previous oral cholera vaccination campaigns by communal section from MSPP (Figure 2, panel B). We geolocated markets during field investigations in Centre.

We calculated Voronoi polygons from estimated point coordinates for each locality. These polygons, or proximity diagrams, consist of all locations closer to the locality point coordinate than any other point coordinate, providing estimated boundaries. We used Voronoi polygons as the basis for all variables to maintain spatial unit consistency and to reduce aggregation bias. We estimated the number of houses per polygon by using a satellite-based house detection shapefile from CNIGS and completed maps by using Google Earth and OpenStreetMap. We multiplied the number of houses by the mean number of household members in the area to estimate the population and incidence rates in each locality (*13*). The distances from each house to an unimproved water source, improved water source, river, and road were calculated and averaged for each polygon. The mean altitude was calculated for each polygon by using raster terrain analysis and rounded to the nearest meter.

### Statistical Analysis

We assessed variables for normal distribution by using histograms, quantile-quantile plots, and Shapiro-Wilk test. For continuous nonnormally distributed variables, we calculated the median and interquartile range (IQR); for categorical variables, we calculated counts and percentages. We categorized continuous variables, including altitude and distances to a road, to an improved water source, to an unimproved water source, and to a river, by using information from quartiles and histograms to form 4 classes. We performed univariate nonparametric statistical tests by using Kruskal-Wallis rank test and Spearman correlation coefficients on cholera incidence and environmental variables. We performed spatial analysis for hotspot detection by using SatScan (https://www.satscan.org).

We performed multistep nonsupervised analysis to classify the localities according to their environmental and spatial characteristics. Hierarchical clustering on the principal components of a multiple correspondence analysis (MCA) was detailed previously (*19*,*20*); we used these data to classify neighborhoods in towns in Haiti (*12*). The first step is an MCA, which is an exploratory method that considers the relationship between variables and reduces complex datasets into fewer dimensions (*21*). We performed MCA by using the original categorical variables and the categorized continuous variables. Active variables included the presence of a market, urban or rural location, vaccination status, and area averaged: altitude, distance to a road, distance to an improved water source, distance to an unimproved water source, and distance to a river. We retained quantitative information only as supplementary variables and did not use these in the determination of the principal components. To reduce basal noise and ensure a more stable classification, we retained the principal components that summarized 95% of the data. We performed hierarchical ascendant classification on the first 16 principal components’ coordinates, which provided classes independent of the number of cholera cases. Then, we compared these classes to cholera cases in a general additive model (GAM) with quasi-Poisson distribution. For spatial autocorrelation, we performed Moran I tests on the number of cases and the GAM residuals. To model spatial dependence, we tested a trend-surface GAM, fitting the geographic location by using 2 dimensional splines on latitude and longitude coordinates, as previously demonstrated (*22*–*24*). We accounted for the increasing population by using an offset of the log population and estimating standardized incidence ratios (SIRs) for each class. We considered p<0.05 statistically significant.

We used QGIS version 2.14.3 (QGIS Development Team, http://qgis.osgeo.org) as a geographic information system (GIS) for mapping. We performed all statistical analyses by using R version 3.3.0 (R Foundation for Statistical Computing, https://www.r-project.org). We used the FactoMineR package in R for classification analysis (*19*) and mgcv for GAMs, with generalized cross validation criteria for smoothing parameter estimations and the gam.check function to verify residual plots (*22*,*25*).

All data remained anonymous with no patient identifiers, in accordance with national and international ethics guidance (*26*). Ethics approval was obtained from the National Bioethics Committee in Haiti, MSPP (reference no. 1516-73).

## Results

**Figure 3:**
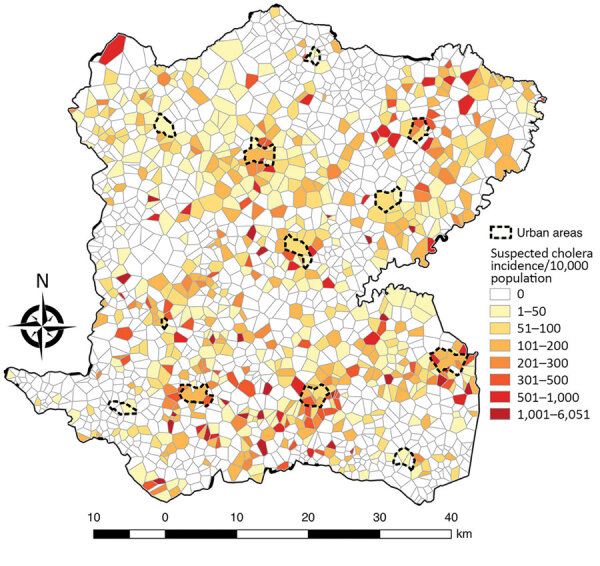
Estimated incidence of suspected cholera cases per 10,000 persons for each locality in Centre Department, Haiti, January 2015–September 2016.

A total of 5,322 suspected cholera cases were recorded in Centre during January 2015–September 2016, and 1,730 localities were identified and mapped. The median locality size was 1.77 km^2^ (IQR 1.15–2.58 km^2^) and median population 300 (IQR 153–530) persons. Among 1,730 localities, 689 (40%) had >1 suspected cholera case (Table 1). The median incidence ratio for all localities was 0 (IQR 0–61.9) per 10,000 persons. Incidence ranged from 0–6,050.9/10,000 persons; 25 localities had an incidence >1,000/10,000 persons (Figure 3). In univariate analysis, the categorical variables statistically significant for incidence were altitude, distance to an unimproved water source, distance to an improved water source, distance to road, distance to a river, presence of market, rural or urban location (p<0.0001), and cholera vaccination (p<0.0001 for all variables) (Table 1).

**Table 1:** Summary descriptive statistics for each locality and variables used for classification analysis used in delineating and analyzing locality-level determinants of cholera, Haiti

Locality information	All localities, n = 1,730	Association with incidence, p value
Median no. suspected cholera cases (IQR)	0 (0–2)	–
Median no. houses (IQR)	65 (33–115)	–
Median estimated population (IQR)	300 (152–530)	–
Median estimated incidence/10,000 (IQR)	0 (0–61.85)	–
Median altitude, m (IQR)*†	360 (264–620)	<0.0001
Median distance to nearest improved water source, m (IQR)*†	842 (464–1,476)	<0.0001
Median distance to nearest unimproved water source, m (IQR) *†	451 (307–702)	<0.0001
Median distance to nearest river, m (IQR)*†	1,263 (554–2,706)	<0.0001
Median distance to nearest road, m (IQR)	638 (185–1,493)	<0.0001
No. markets (%)*	50 (2.9)	<0.0001
No. rural locations (%)*	1,686 (97.5)	<0.0001
No. cholera vaccinations administered (%)*	556 (32.1)	<0.0001

*Active variables used in classification analysis, statistically significant in nonparametric univariate analysis (p<0.05). †Values transformed to categorical variables by using quartiles to form 4 classifications for analysis.

### Hierarchical Clustering on Principal Components

**Figure 4:**
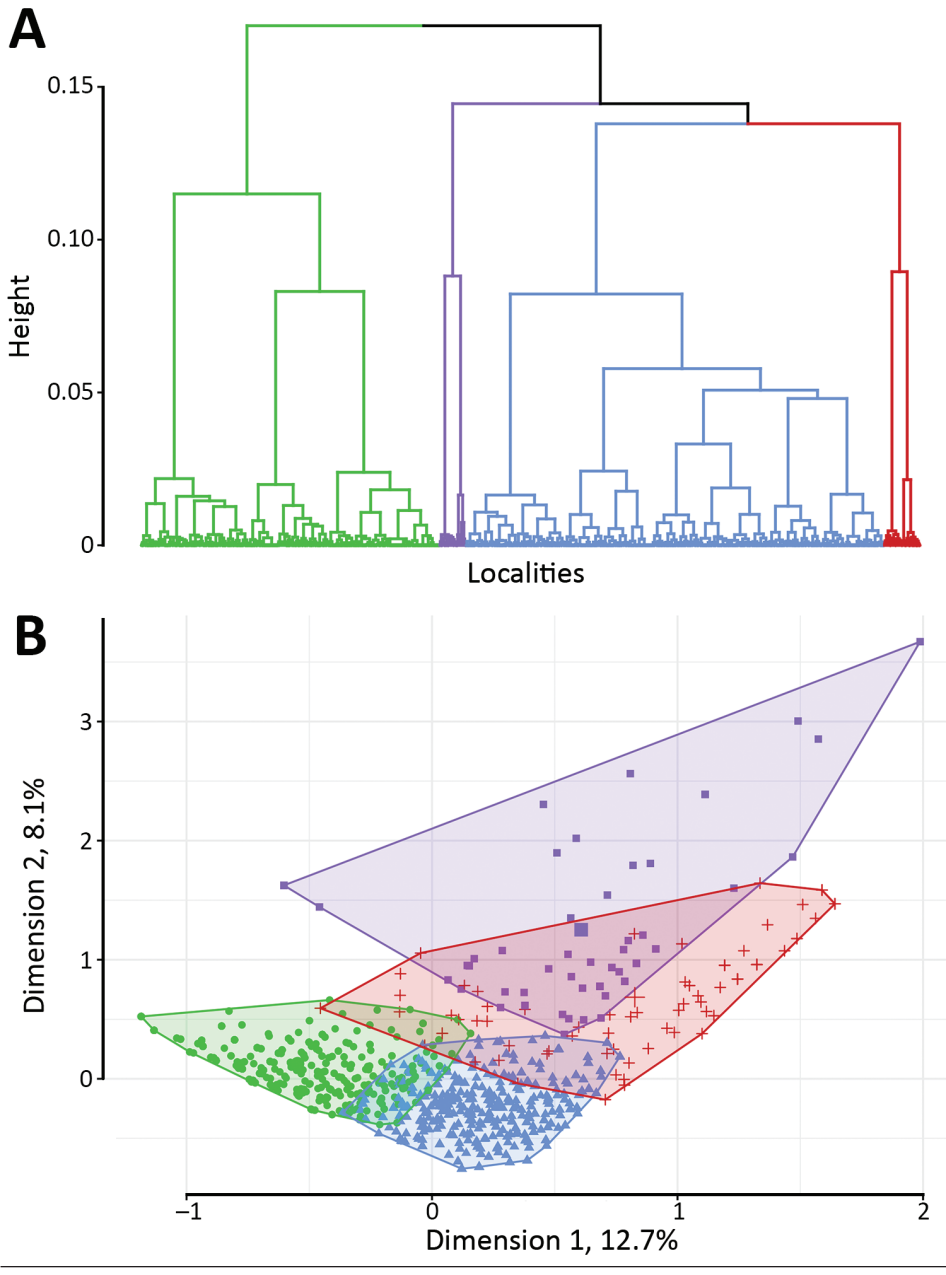
Classification analysis of localities regarding environmental variables based on hierarchical clustering on principal components of multiple correspondence analysis, Centre Department, Haiti. A) Cluster dendrogram demonstrating the division of localities into 4 classes: green, class 1; blue, class 2; purple, class 3; red, class 4. Height indicates the order at which the clusters were joined. B) Factor map demonstrating the 4 classes on the first 2 dimensions of the multiple correspondence analysis with the following variables: altitude, distance to an unimproved water source, distance to an improved water source, distance to road, distance to a river, presence of market, rural or urban, and cholera vaccination. The x and y axes represent the first 2 dimensions of the multiple correspondence analysis; the percentage of the total dataset inertia is represented by each dimension. Each point is a locality, with the shaded areas representing the 4 classes, as in panel A.

**Figure 5:**
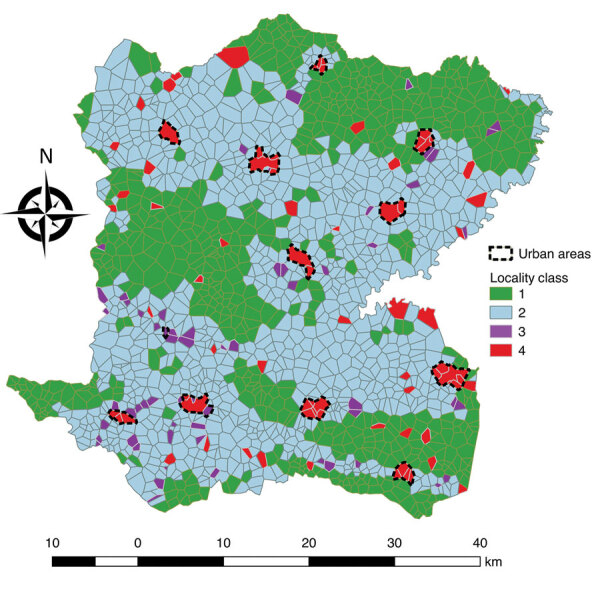
Localities of Centre Department, Haiti, mapped according to hierarchical clustering on principal components classification analysis. Classification analysis determined 2 large lower-risk classes: class 1 localities were remote and higher altitude, and class 2 localities were rural intermediary areas. Two at high-risk classes were identified: class 3 localities are closer to rivers and unimproved water sources, and class 4 localities have markets and are in urban areas.

Hierarchical clustering on MCA provided 4 different classes of localities (Table 2; Appendix Figure 1), demonstrating different environmental and spatial characteristics, which we mapped (Figures 4, 5). The categorical variables that best characterize the portioning into 4 classes were distance to a river, presence of a market, altitude, distance to an unimproved water source, urban or rural location, and distance to a road (p<0.0001 for all).

**Table 2:** Characteristics for each classification identified for analysis of locality-level determinants of cholera, Haiti*

Information for each locality	Class 1, n = 651	Class 2, n = 941	Class 3, n = 61	Class 4, n = 77
Median no. suspected cholera cases (IQR)	0 (0–1)	0 (0–3)	0 (0–3)	6 (1–17)
Median no. houses (IQR)	46 (23–86)	79 (42–127)	52 (26–79)	146 (75–315)
Median estimated population (IQR)	212 (106–396)	364 (194–585)	240 (120–364)	673 (346–1,452)
Median estimated incidence, cases/10,000 population (IQR)	0 (0–15.80)	0 (0–72.31)	0 (0–111)	83.0 (8.6–197.2)
Median altitude, m (IQR)*†	665 (520–816)	287 (232–352)	284 (227–414)	339 (265–461)
Median distance to nearest improved water source, m (IQR)*†	1,213 (665–1,819)	712 (429–1,203)	721 (263–1,357)	338 (233–585)
Median distance to nearest unimproved water source, m (IQR)*†	576 (331–1,080)	439 (317–593)	163 (136–183)	347 (274–533)
Median distance to nearest river, m (IQR)†‡	2,932 (2,047–4,434)	805 (447–1,346)	177 (156–193)	578 (353–1,407)
Median distance to nearest road, m (IQR)†‡	1,544 (882–2,462)	390 (133–816)	210 (70–594)	135 (29–279)
No. (%) markets†	0	0	2 (3)	48 (62)
No. (%) rural locations†	651 (100)	941 (100)	57 (93)	40 (52)
Standardized incidence ratio (95% CI), p value	Referent	1.28 (0.96–1.71), 0.0896	1.71 (1.02–2.87), <0.05	1.69 (1.25–2.29), <0.01

*Class 1 was the reference class, situated at high altitude, and furthest from river and sources. Class 2 was not much more at risk, characterized by a medium distance to roads. Classes 3 and 4 were much more at-risk. Class 3 localities were closer to a river or an unimproved water source and class 4 localities were more often urban with a market. †Active variables used in classification analysis. ‡Transformed to categorical variables for analysis.

Class 1 (n = 621) was most strongly associated with being far from a river, at high altitude, and far from a road (Table 2). Class 1 localities also were remote and farther than average from both unimproved water sources (median 576 m) and improved water sources (median 1,213 m), had no markets, were rural, and had more vaccinated persons.

Class 2 (n = 941) was associated with being a medium distance to the river and at the lowest altitudes (Table 2). Class 2 localities also were associated with being a medium distance from a road, having no markets, being rural, and having more unvaccinated persons. These rural intermediary localities were slightly closer than the average distance to both improved water sources (median 712 m) and unimproved water sources (median 439 m) and lower than average distances to a road (median 390 m).

Class 3 (n = 61) was most notably associated with being closer to rivers (<200 m) and unimproved water sources (<150 m). Class 3 localities also had lower than average distances to a road (median 210 m).

Class 4 (n = 77) was most strongly associated with markets and urban localities. These localities were closer than average to unimproved water sources (median 347 m) and improved (median 338 m) sources and a lower than average distance to a road (median 135 m).

**Figure 6:**
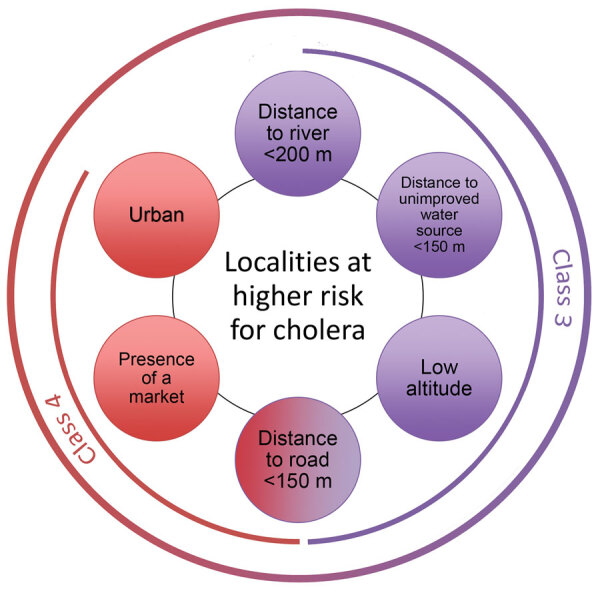
Comparison of the most strongly associated risk factors for cholera between 2 high-risk classes, Centre Department, Haiti. The most strongly associated risk factors for suspected cholera cases were distance <200 m to a river, distance <150 m to an unimproved water source, low altitude, distance to a road <150 m, presence of a market, and urban areas. Class 3 and class 4, both at higher risk for cholera, were close to a road but differed for the other identified risk factors.

Taking class 1 as the reference and taking geographic coordinates and population into consideration, we used a quasi-Poisson GAM to compare the classes and estimate SIRs (Table 2; Appendix). The model confirmed the statistically significantly higher cholera incidence in class 3 (SIR 1.71, 95% CI 1.02–2.87; p = 0.0425) and class 4 (SIR 1.69, 95% CI 1.25–2.29; p = 0.0006). We found class 2 had a slightly increased risk for cholera compared with class 1, with an SIR of 1.28 (95% CI 0.96–1.71), although this difference was not statistically significant at the 5% level (p = 0.0896) (Figure 6).

## Discussion

Our analysis identified 2 main classes of localities at higher risk for cholera: localities close to both rivers (<200 m) and unimproved water sources (<150 m) and urban localities with markets. These criteria are simple ways of identifying high-risk localities for source-based interventions within a large department like Centre. We identified total of 138 higher risk localities, accounting for 8% of all localities.

Reducing population vulnerability to diarrheal illnesses in a low-resource setting has complex challenges, which was confounded in Centre by the large geographic area and difficult terrain. Prioritizing strategies on an easily identifiable number of sources and localities might have implications for preventing cholera outbreaks in Haiti and elsewhere. Whereas previous research emphasized the importance of commune-level data focused on WaSH interventions (*27*), we highlight the importance of fine-scale mapping and using the spatial unit most logical at an operational level. Mapping of localities in Centre Department previously was unavailable, rendering the analysis of case lists impossible. Mapping performed in this study opens doors for further analysis related to cholera and to other health conditions. Furthermore, cholera can be considered as a proxy for vulnerability to waterborne diseases, and targeting these high-risk localities can prevent other disease outbreaks.

Cholera case distribution seems to be linked to geographic patterns, specifically related to environmental hygiene and water source contamination (*28*). Previous studies have demonstrated a substantial association between cholera and populations living in proximity to water bodies (*29*–*32*), lakes (*28*,*33*,*34*), and rivers and to river density (*32*). Increasing elevation has been inversely linked to cholera distribution (*35*). However, due to geographic heterogeneity, the relationship between environmental factors does not fully explain cholera distribution in Haiti. Understanding environmental causation between water sources and cholera is confounded by the difficulties of sampling rivers and water sources and challenges in determining whether these sources are perennial reservoirs or temporary sources of pathogens (*36*,*37*). Other studies have highlighted the change in local populations’ preferences and behaviors to type of water access (*38*,*39*). Furthermore, alongside the proximity to environmental factors, the role of population movement and social factors in the spatial determination of cholera must be considered (*28*,*40*).

Consistent with our results, previous cholera outbreak investigations have reported hotspots in proximity to busy markets (*9*,*12*) and main roads (*32*,*41*), presuming the role of population mobility in creating cholera transmission hubs through the fecal–oral route (*9*,*29*). These social and environmental characteristics must be considered when examining the spatial determinants of cholera in a region. This reinforces the notion that context matters. Our results demonstrate 2 different high-risk classes and highlight the heterogeneous factors across localities, even within the same communal section. We cannot point to a single clear high-risk variable but instead noted patterns of risk depending on the socioenvironmental context of each locality. Our study adds to the literature by using this adaptive contextualized approach at a fine scale across a department, not just limited to smaller urban areas, as seen previously (*9*,*12*).

Numerous reasons can explain an increased cholera risk in localities with markets (*9*). The regular large flux of persons increases the risk for contact with cholera cases. In addition, the poor sanitary conditions of marketplaces increase risk because of inadequate waste disposal, lack of handwashing points, no or poor drainage systems, and the poor hygienic standards of food stalls and latrines, if available. Open defecation areas have been highlighted near Mirebalais market as a source of cholera (*42*). However, the association to markets might be confounded by the geographic proximity of treatment centers, which both often are located in urban areas where a concentration of ill patients aids cholera transmission via contamination of food and water.

Our results highlight the importance of distance to a road in both high-risk classes. Road distance <150 m was statistically significantly associated with class 3 (p<0.0006) and class 4 (p<0.0001). This finding can be interpreted in 2 ways. First, it confirms that cholera is propagated by population movement along main roads. However, main transport hubs also link localities and treatment centers; therefore, cases from localities with easier road access also are more likely to be notified. We used average estimated distances from a locality to a road as an accessible proxy for population mobility, but other information could be used, such as travel time to the nearest town (*43*). Information on actual population mobility by using mobile phone data could be a promising tool to examine spatial spread and improve outbreak preparedness strategies (*40*).

Of note, the association with distance to an improved water source was not a decisive factor in the classification. Increasing distance from an improved water source was associated more with the low-risk class 1, but unimproved water sources were also at a greater median distance. Furthermore, proximity to an improved water source was associated with the high-risk class 4. This finding suggests that the presence of improved water sources in a locality might not fully prevent diarrheal illnesses unless all other potential sources are considered for WaSH improvements. An improved water supply should be available within a 30-minute round trip, according to post-2015 Sustainable Development Goals (*18*), but this availability cannot improve cholera incidence if an unimproved water source is still used for reasons of accessibility and affordability. Our study at the locality level did not consider individualized or household methods of drinking water collection or treatment because no piped water networks are available outside towns and those in towns do not function all the time. Instead, we studied the distance from a house to a water source, including rivers, averaged for the whole locality. However, this variable on geographic accessibility could be confounded by subsequent contamination during travel or storage.

Our study demonstrates the feasibility of fine-scale geographic analysis over a large, mainly rural area to study incidence and spatial epidemiology of cholera in relation to socioenvironmental characteristics and water sources. We used a grassroots approach incorporating expertise from local health and sanitation experts to define a list of localities and comprehensive documentation of water sources. Our approach has potential benefits for future studies by researchers in public health and other disciplines.

As can be common in resource-limited settings with multiple users, the case list required a lengthy data cleaning process. Limitations of the case list include possible missing data from patients who did not go to healthcare centers. However, we believe the case list provided the most comprehensive information available and impressive detail considering the setting constraints.

As demonstrated in previous studies, our field study corroborates that risk for cholera is associated with intertwined socioeconomic and environmental factors and highlights marketplaces located near water bodies and roads in high-density neighborhoods, such as Mirebalais (*29*), as risk factors. By analyzing a wide range of variables, without preceding presumptions on their relationship or correlation, we were able to conduct an exploratory analysis by using MCA. This analysis enabled inclusion of numerous variables despite collinearity and classification of the reduced dimensions in a multivariate model to outline the spatial determinants of cholera.

One limitation of our study was the lack of information regarding excrement management, a recognized predisposing risk factor (*29*,*44*). We attempted to retrieve this information by identifying the location of municipal latrines, but heterogeneous excrement disposal activities make this process futile because open defecation is a common practice (*45*). A recent survey of 13,405 households in Haiti reports only 31% of households have improved, nonshared toilets; in urban areas, 43% have such amenities, but half as many (23%) are found in rural areas (*45*). Another limitation is that trend-surface GAM does not fully address spatial autocorrelation by smoothing the spatial coordinates; however, it does account for trends in geographic data. Nonetheless, trend-surface GAM is a recognized method to model the spatial dependence in the systematic part of the model (*22*–*24*). Furthermore, we were unable to incorporate temporal analyses due to the relatively short timeframe (only 1 dry season). Therefore, we cannot account for all factors associated with cholera incidence, particularly the movement of cholera between localities. In addition, the geographic analysis could not incorporate meteorological information, which is unavailable at the locality level. However, our aim was not to model cholera incidence and dynamics of transmission, which previously has been studied (*46*–*50*). Instead of identifying individual patterns of risk, we used classification analysis to reduce the dimensions of numerous correlated variables to define local-scale risk profiles within a large area affected by cholera. Our study provides information to guide strategies to reduce vulnerability to diarrheal illnesses within a realistic setting, taking the social and environmental context into account.

With no confirmed cholera cases in Haiti since February 2019, the focus in this postepidemic period must be on reducing the vulnerability of the population of Haiti to cholera and other diarrheal illnesses. Our results highlight different typologies of risk at the locality level across a department, defining high risk by access to unimproved water sources and presence of markets in urban localities. Focusing hygiene awareness and prevention strategies in localities with known high-risk factors can help concentrate limited resources and improve efficiency in the fight against future cholera and other waterborne disease epidemics in Haiti and elsewhere.

Appendix Additional information on delineating and analyzing locality-level determinants of cholera, Haiti.
