# Supplementary material for: Delineating and Analyzing Locality-Level Determinants of Cholera, Haiti
Source: Emerg Infect Dis. 2021 Jan;27(1):170–81. doi: 10.3201/eid2701.191787 (PMC7774537; doi:10.3201/eid2701.191787)
Supplement: Appendix — Additional information on delineating and analyzing locality-level determinants of cholera, Haiti. [file 19-1787-Techapp-s1.pdf]

Article DOI: https://doi.org/10.3201/eid2701.191787

Delineating and Analyzing Locality-Level Determinants of Cholera, Haiti

Appendix

SatScan Analysis

Spatial analysis for cholera hotspot detection was computed by using SaTScan version 9.4.2 (*1*). A hotspot was defined as a statistically high-risk cluster in which incidence of suspected cholera cases is higher than in the neighboring areas and was a purely elliptic spatial analysis performed by using a discrete Poisson model. We estimated p values by using Monte Carlo inference (Appendix Table 2).

The results demonstrate a spatial heterogeneity and identified 9 major hotspot ellipses that are comprised of a total of 262 localities. We mapped the location of these 9 hotspot ellipses (Appendix Figure 2), then compared them with the classification of high-risk clusters identified by the hierarchical clustering on principal components (HCPC) methodology. However, due to the diverse differing characteristics of each hotspot, the variables that most contributed to the clustering are not apparent; therefore, interpretation of the numerous clusters was more difficult. We categorized a binary outcome variable by using the SatScan results, so that locality within a hotspot is 1 and locality outside of a hotspot is 0.

Univariate Kruskal-Wallis tests showed a statistically significant difference in cholera hotspots between the 4 classes of localities, $x^2(3) = 18.8$, p = 0.0003. Lower risk classes 1 and 2 and higher risk classes 3 and 4 among localities identified by the HCPC methodology were compared with the binary outcome “hotspot” variable in a binomial general additive model. To model spatial dependence, the geographic location was fitted by using splines as a trend surface and we considered $p<0.05$ statistically significant.

Using lower risk classes as the referent, the general additive model (GAM) showed a statistically significant difference for higher-risk classes being associated with a cholera hotspot.

The GAM confirms the statistically significant higher odds of the higher risk classes being in a cholera hotspot, odds ratio (OR) 3.57 (95% CI 1.63–7.83, $p = 0.001$).

Moran I Inferential Test

Moran I inferential test for spatial autocorrelation was calculated on the raw data, including total number of cases (taking into account the population) and GAM residuals with the spatial coordinates as a smooth term. We used the R package spdep (R Foundation for Statistical Computing, https://www.r-project.org) to compute the Moran I autocorrelation statistic. For the raw total number of cases data, the $p<0.05$ was considered statistically significant ($p = 0.043$) and rejected the null hypothesis of complete spatial randomness, which demonstrates spatial dependence (or autocorrelation) in the raw data.

Thus, we fitted the geographic location by using splines as a trend-surface (2-dimensional spline on spatial projection coordinates). Then we repeated the Moran I test on the GAM residuals, which had a nonstatistically significant p value ($p = 0.055$), seeming to suggest that the model helps to account for the trends of spatial autocorrelation in the data.

Although trend-surface GAM does not fully address the issue of spatial autocorrelation by including a smooth of the spatial locations (projected coordinates), it does account for trends in geographic data (*2*). Trend-surface GAM is a recognized method to model the spatial dependence in the systematic part of the model (*3,4*).

Visual Examples of Locality in Each Class

We provide an example of a locality most representative of each of the 4 main classes of the HCPC (Appendix Figure 3). This paragon helps visualize the differences between each class and provides an example with relevant features. The features portrayed in the figure include the incidence rate (circle size and attributable to incidence rate); area-averaged distance from houses to rivers, roads, improved water sources, unimproved water sources; marketplaces; and vaccinated polygons. Further information, including altitude, is provided in Appendix Table 1.

References

1. Kulldorff M; Information Management Services Inc. SaTScan: software for the spatial, temporal, and space-time scan statistics [cited 2019 March 1]. https://www.satscan.org

2. Cressie NAC. Statistics for spatial data. New York, New York: John Wiley & Sons; 1993.

3. Hastie TJ, Tibshirani RJ. Generalized additive models. New York, New York: Chapman & Hall/CDC; 1990.

4. Wood SN. Generalized additive models, an introduction with R, second edition. Boca Raton, FL: Chapman & Hall/CDC; 2006.

Appendix Table 1. Univariate analysis, nonparametric Spearman correlation coefficients, and p values used to delineate and analyze locality-level determinants of cholera, Haiti

Variable	Variable	Correlation coefficient	p value*
Incidence	Mean altitude	−0.219877577	0
Incidence	Distance to a road	−0.231237548	0
Incidence	Distance to unimproved water source	−0.118602227	<0.0001
Incidence	Distance to improved water source	−0.185149843	<0.0001
Incidence	Distance to river	−0.147558669	<0.0001

*Quantitative variables.

Appendix Table 2. List of cholera hotspots detected by SaTScan, Haiti*

High-risk hotspot	Relative risk	p value
1	2.15	<0.0001
2	2.81	<0.0001
3	6.53	<0.0001
4	2.10	<0.0001
5	14.60	<0.0001
6	21.42	<0.0001
7	16.20	<0.0001
8	9.73	<0.0001
9	7.85	0.0002
10	3.67	0.999

*M. Kulldorff, https://www.satscan.org (*1*).

Appendix Table 3. Variables used to create visualization of representative localities for each class in a spatial analysis of cholera, Haiti

	Class 1 locality	Class 2 locality	Class 3 locality	Class 4 locality
Variables for each locality	Nan Chambo	Palmari	Moro	Cerca la Source Ville
No. suspected cholera cases	1	1	7	34
No. houses	65	113	41	1,117
Estimated population	300	521	189	5,149
Estimated incidence/10,000 persons	33.37	19.20	370	66
Altitude, m in meters*†	764	414	224	376
Distance to nearest improved water source, m*†	2,194	385	675	155
Distance to nearest unimproved water source, m*†	154	455	189	292
Distance to nearest river, m*†	2,073	575	189	508
Distance to nearest, m*†	4,785	1,778	1,106	9
Presence of market *	No	No	No	Yes
Rural locality*	Yes	Yes	Yes	No

*Active variables used in classification analysis.
†Transformed to categorical variables for analysis.

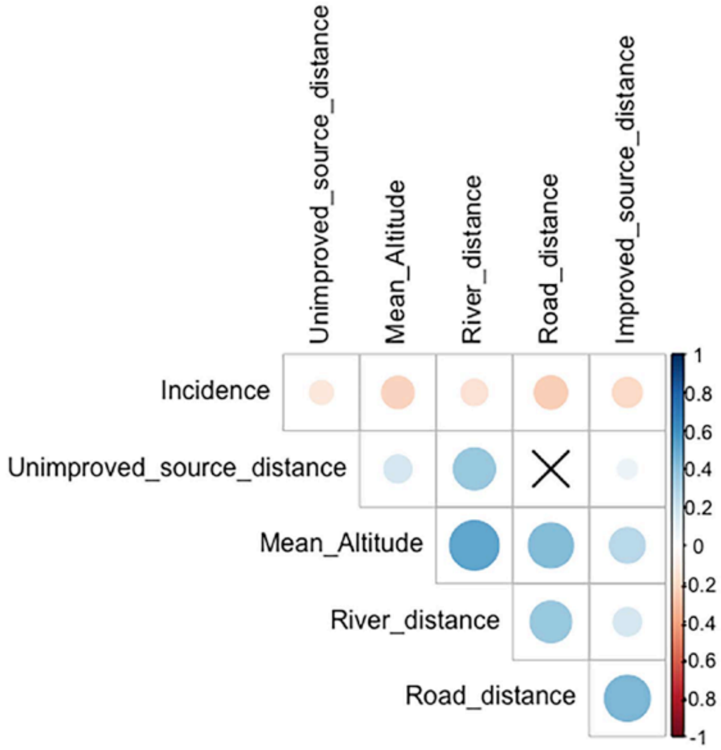

Appendix Figure 1. Correlation matrix of factors associated with high risk for cholera, Centre Department, Haiti. We used Spearman correlation coefficients to perform univariant analysis on cholera incidence and environmental variables. Blue indicates positive correlations (i.e., lower risk for cholera) and red indicates negative correlations (i.e., higher risk for cholera). Color intensity and the size of the circle are proportional to the correlation. Legend color shows the correlation coefficients and the corresponding colors. X indicates non-significative result.

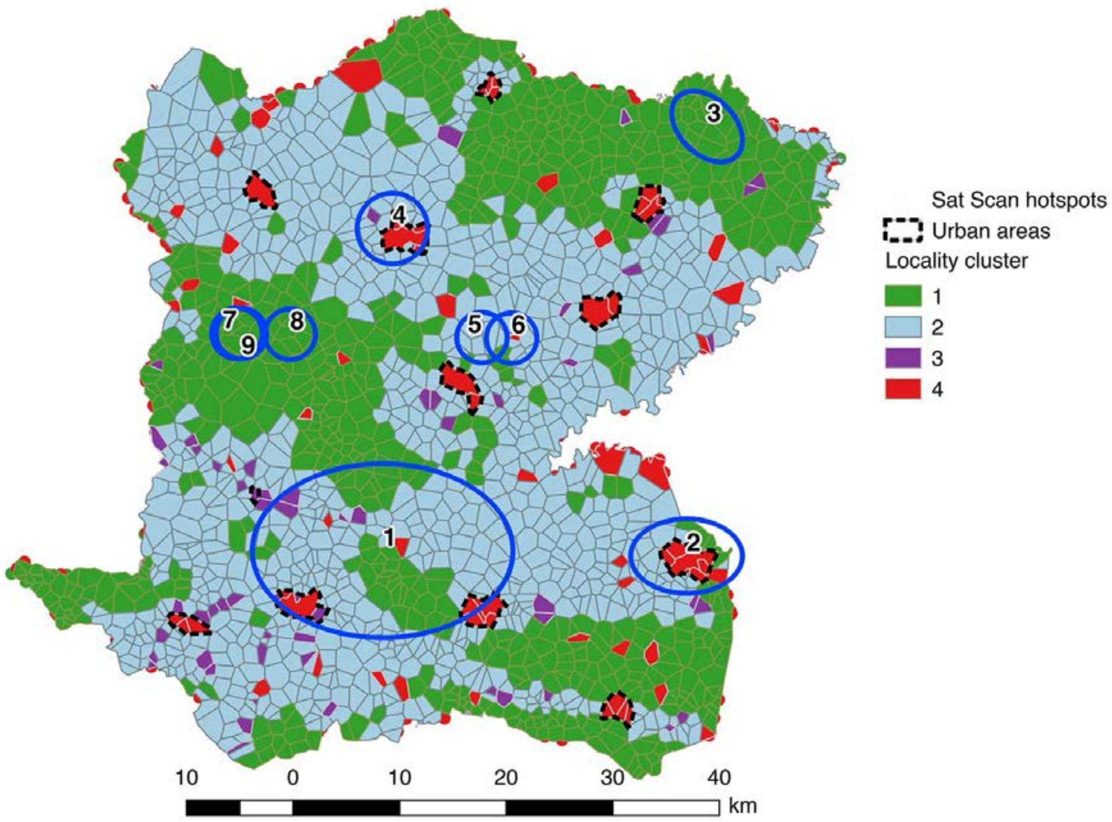

Appendix Figure 2. Locality map demonstrating the 4 classes identified by hierarchical clustering on principal components, Centre Department, Haiti. Numerals in blue circles indicate the 9 cholera hotspots identified by SatScan.

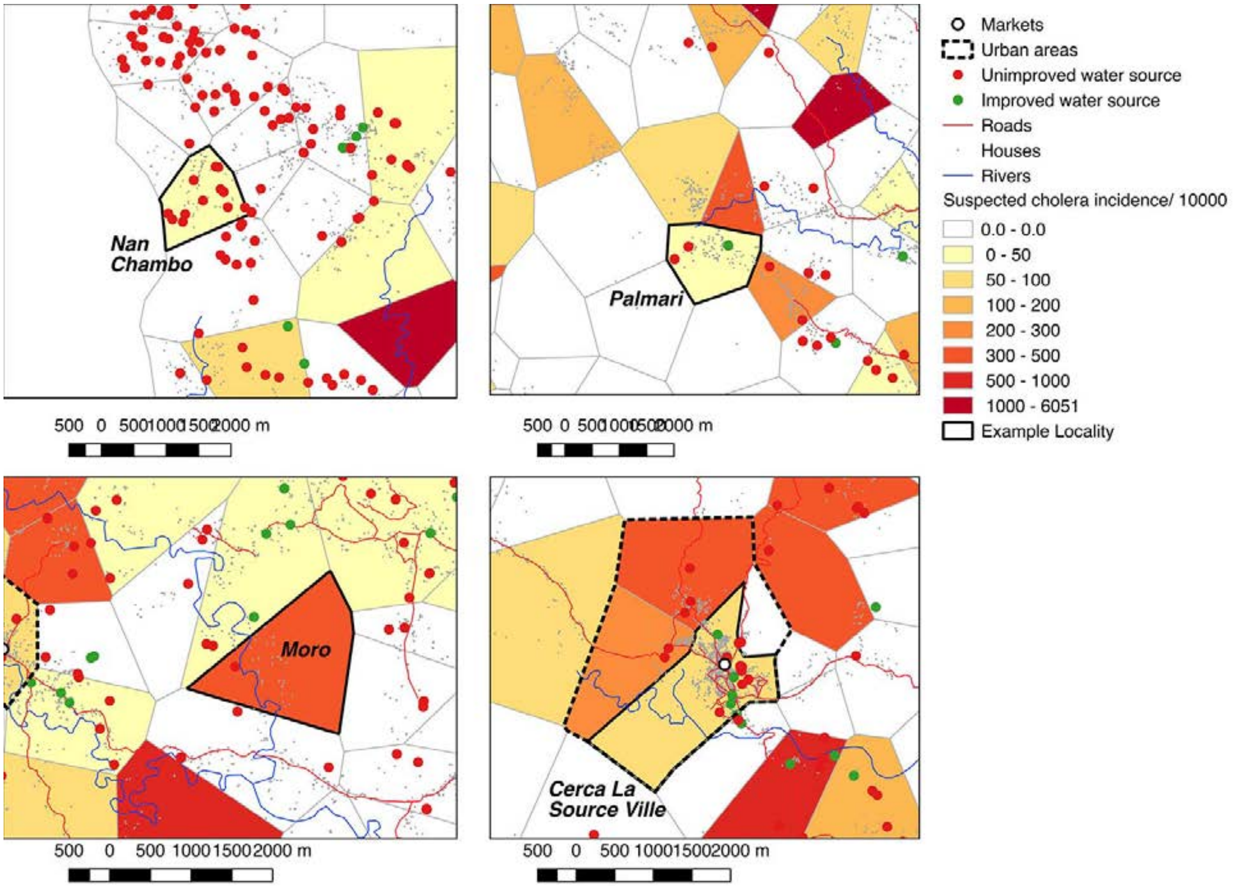

Appendix Figure 3. Maps demonstrating fine scale data on factors associated with risk for cholera, Centre Department, Haiti. Nan Chambo (top left) is a remote class 1 locality at high altitude, far from rivers and roads; Palmari (top right) is a rural intermediary class 2 at lower altitude and a medium distance from a river; Moro (lower left) a class 3 locality close to rivers and unimproved water sources; and Cerca La Source Ville (lower right) is a class 4 urban locality that has markets.
